# Upregulation of Wnt2b exerts neuroprotective effect by alleviating mitochondrial dysfunction in Alzheimer's disease

**DOI:** 10.1111/cns.14139

**Published:** 2023-02-27

**Authors:** Ling‐Zhi Xu, Bing‐Qiu Li, Fang‐Yu Li, Mei‐Na Quan, Wei Qin, Ying Li, Wen‐Wen Li, Yu Zhao, Yi‐Ping Wei, Jian‐Ping Jia

**Affiliations:** ^1^ Innovation Center for Neurological Disorders and Department of Neurology, Xuanwu Hospital Capital Medical University, National Clinical Research Center for Geriatric Diseases Beijing China; ^2^ Beijing Key Laboratory of Geriatric Cognitive Disorders Beijing China; ^3^ Clinical Center for Neurodegenerative Disease and Memory Impairment Capital Medical University Beijing China; ^4^ Center of Alzheimer's Disease, Beijing Institute of Brain Disorders, Collaborative Innovation Center for Brain Disorders Capital Medical University Beijing China; ^5^ Key Laboratory of Neurodegenerative Diseases, Ministry of Education Beijing China; ^6^ Cell Therapy Center, Beijing Institute of Geriatrics, Xuanwu Hospital Capital Medical University National Clinical Research Center for Geriatric Diseases Beijing China

**Keywords:** Alzheimer's disease, mitochondrial function, neuronal damage, Wnt2b

## Abstract

**Aims:**

This study investigated the relationship between plasma Wnt2b levels and Alzheimer's disease (AD), and explored the effect of Wnt2b on mitochondrial dysfunction in AD.

**Methods:**

Healthy and AD subjects, AD transgenic mice, and in vitro models were used to investigate the roles of Wnt2b in abnormalities in canonical Wnt signaling and mitochondria in AD. RT‐qPCR, immunoblotting, and immunofluorescence analysis were performed to assay canonical Wnt signaling. Mitochondrial structure was analyzed by electron microscopy. Flow cytometry was used to examine the intracellular calcium and neuronal apoptosis.

**Results:**

Plasma Wnt2b levels were lower in AD patients and positively correlated with cognitive performance. Similarly, Wnt2b was reduced in the hippocampus of AD mice and in vitro models. Next, Wnt2b overexpression and recombinant Wnt2b were used to endogenously and exogenously upregulate Wnt2b levels. Upregulation of Wnt2b could effectively prevent downregulation of canonical Wnt signaling, mitochondrial dysfunction in in vitro AD models. Subsequently, intracellular calcium overload and neuronal damage were ameliorated.

**Conclusions:**

Our study highlights that Wnt2b decline is associated with cognitive impairment in AD, and upregulation of Wnt2b can exert neuroprotective effects in AD, particularly in ameliorating mitochondrial dysfunction.

## 1 INTRODUCTION

Alzheimer disease (AD) is the most common neurodegenerative disorder leading to dementia in the elderly worldwide. AD is neuropathologically characterized by the presence of extracellular β‐amyloid plaques and intracellular neurofibrillary tangles composed of hyperphosphorylated tau protein.^1^ Wnt signaling participates in multiple aspects of cellular function, and contributes to synapse formation, synaptic activity, and neurogenesis in the brain,^2^, ^3^, ^4^ and dysfunction of Wnt signaling may aggravate the pathogenesis and progression of AD.^5^

We have been focusing on the research and development of blood and cerebrospinal fluid (CSF) markers that can be used for early diagnosis of AD, and we have found a variety of proteins that were significantly different in the blood and CSF of AD patients and cognitively normal subjects.^6^, ^7^ Recently, we found that Wnt2b, a member of WNT family, in the plasma of AD patients are lower than in healthy controls. Wnt2b has been studied in cancers or tumor‐related diseases,^8^ while Wnt2b is one of the most important components that can induce the particular developing pattern of the fetal hippocampus and related to neurogenesis, such as retinal ganglion cells.^9^, ^10^ Interestingly, Wnt2b is also characterized as a mitochondria‐expressed protein which shuttles between mitochondria and the nucleus.^11^, ^12^ However, Wnt2b has not been studied in AD, particularly since it is expressed in mitochondria, unlike other Wnt family members. The above evidence makes us intend to further explore the roles of Wnt2b in AD, especially on mitochondrial dysfunction. As known, mitochondrial dysfunction contributes to the development and progression of the pathological process that underlies AD, especially at the early stage.^13^, ^14^ Some mitochondria‐targeting drugs have shown significant effect in improving pathological change and cognitive impairment in AD model.^15^ Therefore, we hypothesized that upregulation of Wnt2b may improve mitochondrial dysfunction in AD.

In the present study, we were unable to detect CSF Wnt2b levels due to lack of appropriate CSF Wnt2b kit and inadequate detection techniques unfortunately, so human samples were only tested in plasma. We consider Wnt2b in plasma mainly from peripheral and cell secretion in blood. There are also some Wnt2b in plasma that may be neurogenic, and molecules in the plasma might be derived from central nervous system and these molecules can enter the periphery through exosomes and other ways.^16^ Many studies have suggested that plasma biomarkers can reflect the progress and situation of neurological disorders to a certain extent.^17^ Moreover, plasma is a more readily available and simple sample than CSF and brain tissue and has better translational potential. In this study, we also analyzed the correlation between plasma Wnt2b and cognitive function. In neurological disorders, some molecules in plasma, including proteins related to neurogenesis or synaptic plasticity, often have similar changes to those in the brain.^18^ Therefore, we want to explore whether the changes in Wnt2b in the brain are similar to those in the plasma of AD patients. Considering the difficulty of detecting in the brain of AD patients, we examined the levels of Wnt2b protein in the brain of AD mice at different months of age and analyzed whether the changes were similar to those of plasma Wnt2b. We next investigate the effect of upregulating Wnt2b on mitochondrial dysfunction and neuronal damage in in vitro AD model. Our findings will provide some basis for the protection of Wnt2b in mitochondrial function in AD and highlight its potential for clinical application and drug development for AD.

## 2 MATERIALS AND METHODS

### 2.1 Reagents and antibodies

Synthetic human Aβ_1‐42_ peptide was purchased from GL Biochem , Dulbecco's Modified Eagle's Medium (DMEM), fetal bovine serum (FBS), penicillin, and streptomycin were purchased from Gibco , mouse recombinant Wnt2b (rWnt2b) was purchased from Cusabio, 1,1,1,3,3,3‐Hexafluoro‐2‐propanol (HFIP) was purchased from Sigma‐Aldrich . The primary antibodies used were rabbit anti‐Wnt2b (ab178418; Abcam), mouse anti‐β‐catenin (ab32572; Abcam); rabbit anti‐phospho‐β‐catenin (Thr41/Ser45) (9565 S; Cell Signaling Technology); rabbit anti‐GSK3β (ab32391; Abcam); rabbit anti‐phospho‐GSK3β (Ser9) (ab75814; Abcam); rabbit anti‐BDNF (ab108319; Abcam); rabbit anti‐SDHB (A10821; ABclonal); mouse anti‐β‐actin (ab6276; Abcam); The secondary antibodies used were Goat Anti‐Rabbit IgG (HRP) (ab205718; Abcam); Goat Anti‐Mouse IgG (HRP) (ab6789; Abcam); Donkey anti‐Rabbit IgG (ab150073; Abcam).

### 2.2 Animals and hippocampal protein extraction

PS1 V97L mice aged at 6 and 9 months, and their sex‐ and age‐matched wildtype (WT) littermates were enrolled in this study. PS1 V97L Tg mice expressing human PS1 harboring the V97L mutation were generated as previously described.^19^, ^20^ The study protocol was approved by the Ethics Committee of Capital Medical University (No. AEEI‐2017‐004). These mice were housed under standard conditions. The brains of PS1 V97L transgenic mice and wild‐type mice were extracted and the hippocampus were then isolated and homogenized after being lysed with RIPA lysis buffer with protease inhibitor and a phosphatase inhibitor mixture (Applygen Technology) for 30 min. The protein concentrations of all of the samples were determined using the BCA assay kit (Applygen Technology). The protein concentration of the samples was normalized and then the samples were denatured at 98°C for 10 min.^21^

### 2.3 Preparation of AβOs

Oligomerized Aβ_1–42_ was prepared as previously reported.^22^ Briefly, Aβ_1–42_ was dissolved at 1 mg/mL in HFIP, and the HFIP was removed in a SpeedVac vacuum concentrator after 1 h. The peptide film was resolved at 1 mM in DMSO (Sigma‐Aldrich). The solution was diluted to 100 μM with DMEM. Then, the solution was incubated at 4°C overnight followed by centrifuging at 14,000 × *g* for 10 min at 4°C to remove any insoluble aggregates.^23^

### 2.4 Cell culture and transfection

HT22 cells were cultured in DMEM medium supplemented with 10% FBS, 50 U/mL penicillin, and 50 g/mL streptomycin solution, and maintained in a humidified atmosphere of 5% CO2 and 95% air at 37°C. Cells were treated with 5 μM AβOs and 300 ng/mL recombinant Wnt2b (rWnt2b) for 24 h. The pcDNA3.1 constructs with WNT2B overexpression (pcDNA3.1‐WNT2B) were designed and synthesized by Sangon Biotechnology Company, and pcDNA3.1 was served as the normal control (NC). The transfections of pcDNA3.1‐WNT2B or pcDNA3.1 were performed in HT22 cells using Lipofectamine 3000 reagent (Invitrogen) by following the manufacturer's instructions. At 24 h following transfections, cells were treated with 5 μM AβOs for another 24 h, and cells were processed for further in vitro assays.

### 2.5 Immunoblotting

The western blot assays were based on our previous studies.^21^, ^24^ The samples were subjected to SDS‐polyacrylamide gel electrophoresis for about 40 min at 80 V in stacking gel and about 50 min at 120 V in resolving gel. Proteins were transferred electrophoretically to PVDF membranes (Millipore) at 250 mA for 1–2 h. Membranes were washed with TBST (Tris‐Buffered Saline plus 0.05% Tween‐20, pH 7.4) and then incubated in blocking buffer (5% skimmed milk in TBST) for 1 h at room temperature. The membranes were incubated overnight at 4°C on a shaker with primary antibody in TBST plus 5% skimmed milk. After four 5‐min washes in TBST buffer, the membranes were incubated for 1 h at room temperature on a shaker with horseradish peroxidase (HRP)‐conjugated secondary antibody diluted in blocking buffer. The membranes then underwent four 5‐min washes with TBST and were visualized using the ECL chemiluminescence detection kit (Millipore). The immunoblots were quantified with the Gel Doct EZ system (Bio‐Rad). Band intensities were normalized to β‐actin and were analyzed with Quantity One software (version 4.4.0, BioRad).

### 2.6 RT–qPCR assay

Total RNA was extracted from HT22 cells using the RNAsimple Total RNA kit (Tiangen). After DNAse digestion reverse transcription of total RNA was performed using FastKing RT Kit (Tiangen). 2x TB Green Premix Ex Taq II (Takara) was used for real‐time quantitative polymerase chain reaction (RT‐qPCR) of the respective genes. Cycling conditions were as follows: initial denaturation at 95°C for 30 s, followed by 40 cycles of 95°C for 5 s and 60°C for 30 s. For amplification the following primers were used (5′ > 3′orientation): for *WNT2B*: (fw: GACACGTCCTGGTGGTACATA; rev: GGCACTCTCGGATCCATTCC); for *CLPX‐1*: (fw: TGTTGGCCAGTCGTTTGCTA; rev: CCCGTCTTCTTATTTCTAACTCTCT); for *OMA1*: (fw: CGTCAATGCCTTTGTGCTCC; rev: CTAGCCTTTTCTGCGGCGT); for GAPDH: (fw: TGCACCACCAACTGCTTAG; rev: GATGCAGGGATGATGTTC); Specificity of PCR products was confirmed by analysis of a melting curve. RT‐qPCR amplifications were performed on a StepOne Real‐Time PCR system (Applied Biosystems) and all experiments were done in duplicate. In all experiments, the relative quantification of gene expression was achieved by the ΔΔCt method as described.

### 2.7 Immunofluorescent staining

The mouse was perfused with 0.01 mol/L PBS and 4% paraformaldehyde (pH 7.4), followed by post‐fixation overnight at 4°C. The cultured cells were washed with PBST three times, fixed with 4% paraformaldehyde for 20 min, and followed by permeabilization with 0.1% Triton X‐100. After blocked with 10% donkey serum (Solarbio), cells were incubated with primary antibody. Primary antibody incubation was performed on a shaker overnight at 4°C, followed by secondary antibody incubation for 1 h at room temperature. The following antibodies were used at the indicated concentrations: anti‐Wnt2b antibody (1:100), donkey anti‐rabbit IgG Alexa Fluor Plus 488 secondary antibody (1:500). DAPI reagent (Beyotime) was used for the nucleus staining. Images were taken using a fluorescence microscope (Olympus) and processed with ImageJ (https://imagej.nih.gov/ij/).

### 2.8 Mitochondrial structure and function

Mitochondrial structure was analyzed by electron microscopy. Then, images were obtained using a digital video camera (Tecnai G20 TWIN, FEI company), and the images were transferred to a computer. The intracellular ATP levels were analyzed by ATP assay kit (Beyotime Institute of Biotechnology). The mitochondrial membrane potential was detected by JC‐1 fluorescent dye kit (Beijing Solarbio Science & Technology Co., Ltd). Cells were subjected to JC‐1 fluorescent dye for 20 min in the dark. Fluorescence microscope was used to capture images, and the ratio of red fluorescence intensity to green fluorescence intensity was calculated to analyze the change in mitochondrial membrane potential. For ROS detection, cells were subjected to 1 μM DCFH‐DA (Beijing Solarbio Science & Technology Co., Ltd) at 37°C for 20 min. Fluorescence microscope was then used to capture images (Ex: 488 nm, Em: 525). Fluorescence of the control group was defined as 100% and fluorescence of other groups was normalized to that of control group.

### 2.9 Flowcytometry

Apoptosis and intracellular Ca^2+^ fluctuation were assayed by flow cytometry (BD FACS Calibur, BD Biosciences) as previous described.^25^ Apoptosis was measured using Annexin V‐FITC Apoptosis Detection Kit (C1062, Beyotime) according to the manufacturer's guidelines. Fluctuation of the intracellular Ca^2+^: HT22 cells were washed twice with Ca^2+^ − and Mg^2+^ − free Hank's Balanced Salt Solution (HBSS), and digested with 0.05% trypsin, and rinsed with HBSS before resuspended in Fluo4‐AM at the final concentration of 5 mol/L, and then incubated 20 min in 37°C with darkness. After incubation, the cell suspension was centrifuged at 1000 rpm for 5 min and precipitate was washed with HBSS three times then the final cell suspension was collected and analyzed with FlowJo software (TreeStar). The fluorescence intensity in each group was recorded, which reacted to the intracellular free Ca^2+^ concentration.

### 2.10 Human subjects and plasma Wnt2b detection

40 normal cognitive (NC) subjects and 40 AD patients were recruited from Xuanwu Hospital in Beijing and the study was approved by the Ethic Committee for the Conduct of Human Research at Xuanwu Hospital, Capital Medical University (No. LYS [2017] 004). The demographic characteristics of AD and NC subjects, including age, gender, education, Montreal Cognitive Assessment (MoCA), APOE ε4 status, were recorded (Table 1). Non‐fasting plasma samples were collected in tubes that contained ethylenediamine tetra‐acetate (EDTA) and stored at −80°C for further analysis. MoCA is extensively used to evaluate cognitive impairment. The concentration of plasma Wnt2b was measured using commercially available Human Wnt2b (WNT2B) ELISA kit (CSB‐EL026134HU, Wuhan, China).^26^ For the detection of Wnt2b protein, the plasma was directly detected with stock suspension, and its concentration was presented as pg/mL through a standard curve.

**TABLE 1:** Characteristics of NC subjects and AD subjects

	NC (*N* = 40)	AD (*N* = 40)	*t*/Chi‐square	*p* value
Age (Years)	64.42 ± 3.10	65.70 ± 5.53	−1.272	0.208
Male (*n*, %)	23 (57.5)	20 (50)	0.453	0.501
Education (Years)	14.30 ± 2.76	9.54 ± 4.47	5.74	<0.001
MoCA	28.22 ± 1.27	10.78 ± 5.85	18.434	<0.001
*APOE ε4* (Number, %)	5 (12.5)	21 (52.5)	15.289	<0.001
Wnt2b (pg/mL)	167.76 ± 82.75	132.56 ± 52.17	2.276	0.026

*Abbreviations*: AD, Alzheimer's disease; MoCA: Montreal Cognitive Assessment; NC: normal cognitive.

### 2.11 Statistical analysis

Data were presented as the mean ± standard error of mean (SEM) or mean ± standard deviation (SD) or number (%). The biochemical measurements were performed with the experimenter blind to the experimental groups. Statistical analysis of data were performed using PRISM (version 8) (GraphPad Software) and/or SPSS for Windows (version 21.0) (SPSS Inc.). The difference between groups was performed using χ^2^ test for categorical variables. All continuous data were subject to test for normality. Variables subject to normal distribution were analyzed using independent samples *t*‐test, or one‐way analysis of variance (ANOVA) followed by Tukey's post hoc test as appropriate. Variables that do not exhibit a normal distribution were analyzed using Mann–Whitney U test or Kruskal‐Wallis test. The multiple logistic regression was performed to detect the influence of Wnt2b on AD after adjusting for age, gender, APOE ε4, and education with SPSS software. The association between qualitative variables was evaluated by Spearman correlation. Values of *p* < 0.05 were considered statistically significant.

## 3 RESULTS

### 3.1 Plasma Wnt2b was decreased in AD patients and correlated with cognitive function

**FIGURE 1:**
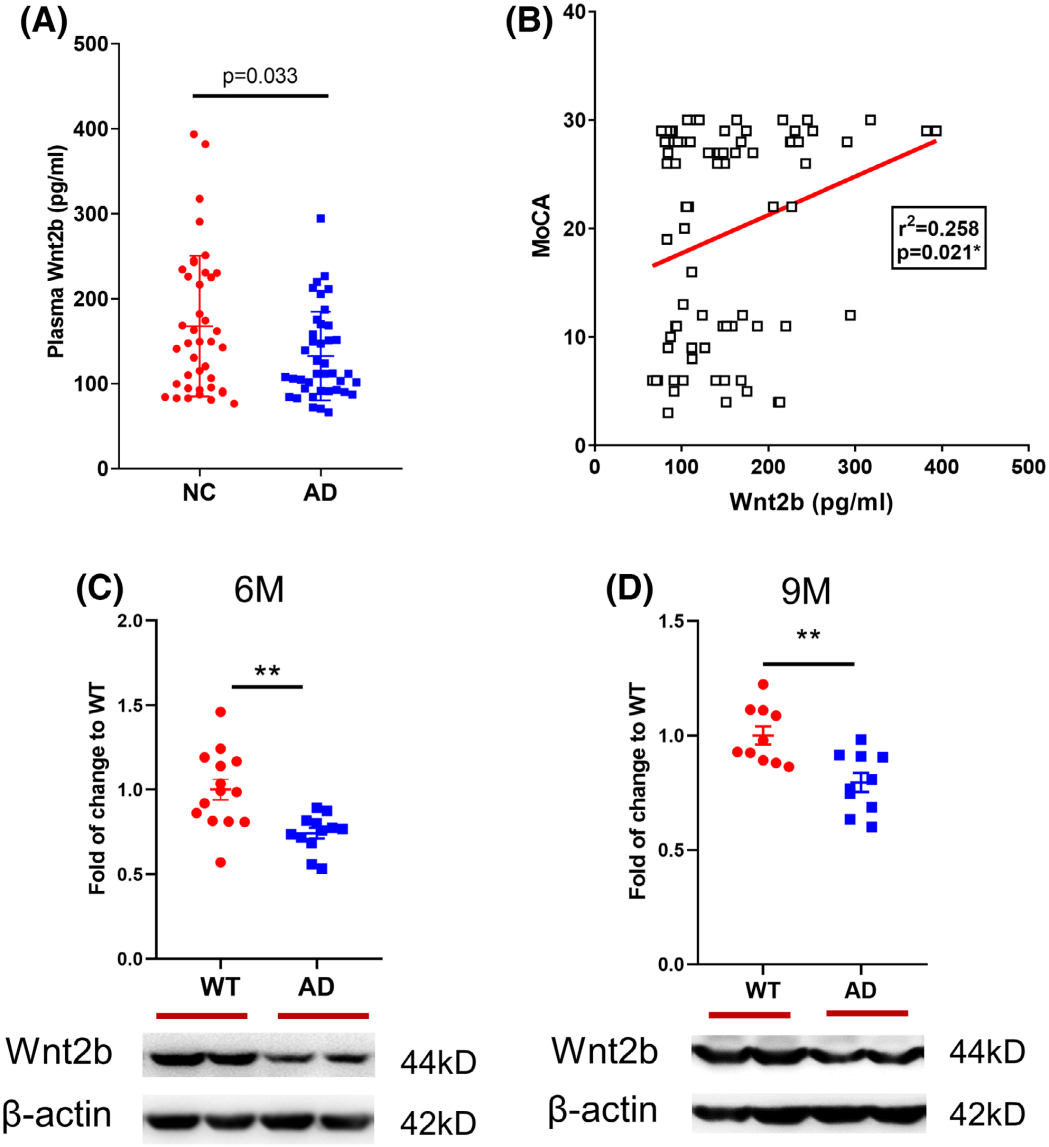
Wnt2b was decreased in plasma of AD patients and in brain of AD mice. (A) Plasma Wnt2b levels in AD patients were lower than that in NC groups. (B) Plasma Wnt2b protein was positively correlated with cognitive function (*r* ^2^ = 0.258, *p* = 0.021). (C) Representative western blots and quantification of fold changes in Wnt2b, β‐Actin expression in the hippocampus of 6‐month‐old mice. (D) Representative western blots and quantification of fold changes in Wnt2b, β‐Actin expression in the hippocampus of 9‐month‐old mice. Data values are expressed as mean ± SD or mean ± SEM. The difference between groups was performed using χ^2^ test. The multiple logistic regression was performed to detect the influence of Wnt2b on AD after adjusting for age, gender, APOE ε4, and education with SPSS software. The association between qualitative variables was evaluated by Spearman correlation. The difference between WT and AD mice was performed using independent samples *t*‐test. *p < 0.05 in comparison to WT mice, **p < 0.01 in comparison to WT mice. AD, Alzheimer's Disease; NC, normal cognitive; WT, wild‐type

We have previously reported a variety of proteins that have significant changes in the blood or cerebrospinal fluid (CSF) of AD patients.^6^, ^7^ Recently, we found that plasma Wnt2b level in AD patients (132.6 ± 52.17 pg/mL) was lower than that of NC subjects (167.8 ± 82.75 pg/mL; Figure 1A), and demographic data as shown in Table 1. After other confounders including age, gender, education, and *APOE ε4* were adjusted, there were still differences between NC and AD subjects in plasma Wnt2b level (Table S1). Further correlation of Wnt2b protein and cognitive performance (MoCA) was analyzed, and plasma Wnt2b was positively correlated with MoCA scores (Figure 1B).

### 3.2 Wnt2b expression was declined in in vivo and in vitro AD model

**FIGURE 2:**
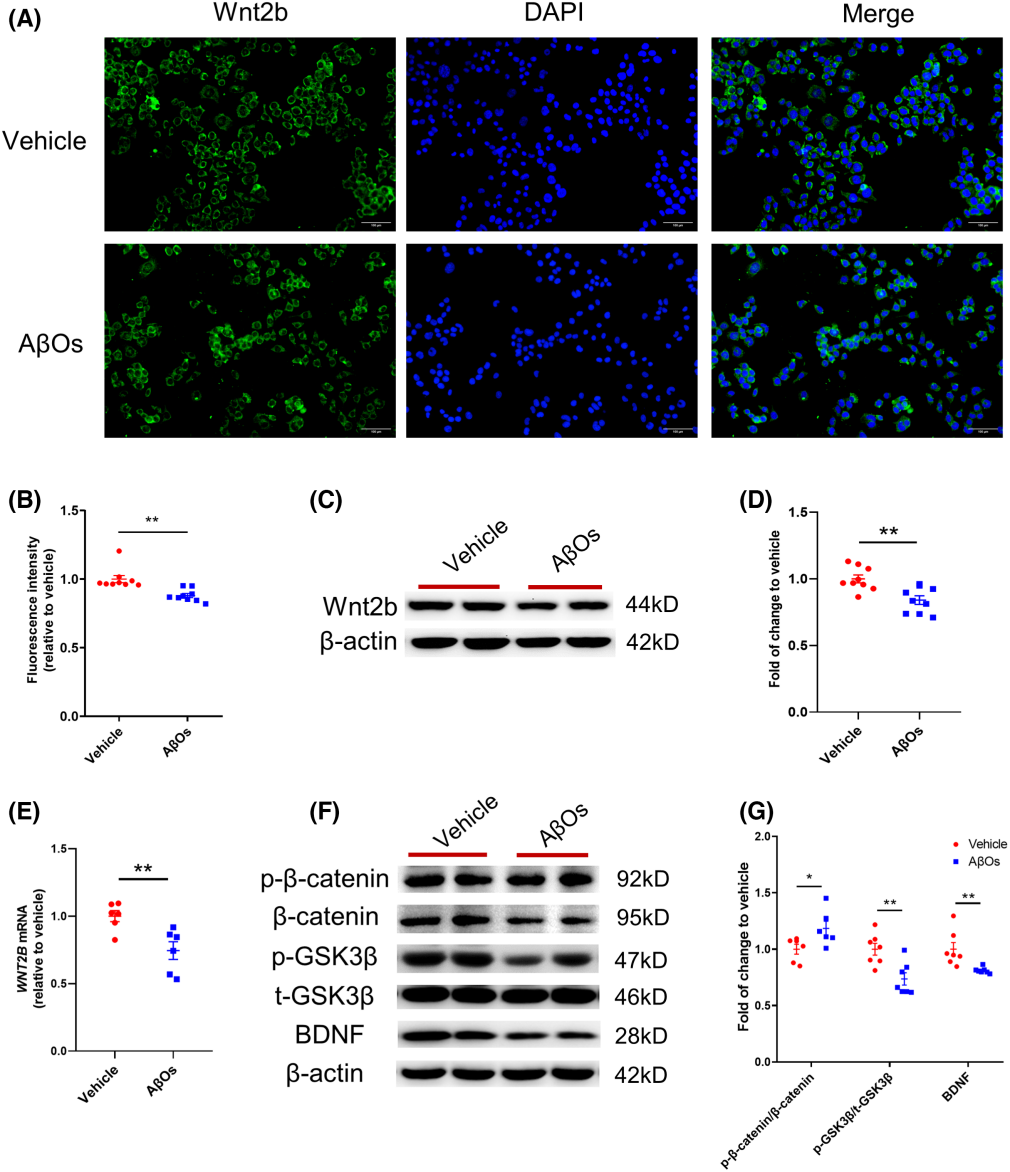
Wnt2b was related to downregulation of canonical Wnt signaling in the presence of Aβ oligomers (AβOs) (A) Representative immunofluorescent image of Wnt2b, DAPI in HT22 cells. (B) Quantification of fold changes in fluorescence intensity of Wnt2b in HT22 cells relative to vehicle after AβOs treatment. (C and D) Representative western blots and quantification of fold changes in Wnt2b expression in HT22 cells relative to vehicle after AβOs treatment. (E) Quantification of fold changes in *WNT2B* expression in HT22 cells relative to vehicle after AβOs treatment. (F) Representative western blots of p‐β‐catenin, β‐catenin, p‐GSK3β, total p‐GSK3β, BDNF, β‐Actin expression in HT22 cells. (G) Quantification of fold changes in p‐β‐catenin/β‐catenin, p‐GSK3β/GSK3β, BDNF in HT22 cells relative to vehicle after AβOs treatment. Data values are expressed as mean ± SEM. The difference between groups was performed using independent samples *t*‐test. *p < 0.05 in comparison to vehicle, **p < 0.01 in comparison to vehicle

In order to figure out Wnt2b expression and levels in the brain, we used PS1 V97L transgenic mice that an AD transgenic model mice constructed and reported by our team. PS1 V97L mice began to appear Aβ oligomers in the brain at the age of 6‐month and showed cognitive impairment at 9‐month‐old.^19^, ^27^ 6‐ and 9‐month‐old PS1 V97L mice represented preclinical AD and clinical AD respectively in this study. Blotting analysis verified the expression of Wnt2b in the hippocampus of PS1 V97L mice was reduced (Figure 1C). And similar results were found in the 9‐month‐old PS1 V97L mouse (Figure 1D). Further in vitro study was performed in mouse hippocampal HT22 cells treated with AβOs. Treatment with 5 μM AβOs in HT22 cells induced significant neuronal damage at 24 h (Figure S1). After hippocampal cells treated with 5 μM AβOs for 24 h, immunofluorescence intensity of Wnt2b (Figure 2A,B) and Wnt2b expression in blotting analysis (Figure 2C,D) were decreased. And *WNT2B* mRNA in HT22 cells after AβOs exposure was declined compared to vehicle group (Figure 2E). Besides, the ratio of p‐GSK3β (Ser9) to total GSK3β and BDNF level were reduced, and the ratio of phosphorylated β‐catenin (Thr41/Ser45) to total β‐catenin was increased in canonical Wnt signaling in the presence of AβOs (Figure 2F,G).

### 3.3 Overexpression of *WNT2B* improved mitochondrial dysfunction and neuronal damage in HT22 cells in the presence of AβOs

In addition to being involved in canonical Wnt signaling, Wnt2b is characterized as a mitochondrial protein.^11^, ^28^ We found that Wnt2b was both expressed in the mitochondrial and cytoplasm fraction of mouse hippocampal HT22 cells (Figure S2), and mitochondrial dysfunction is an important pathological feature of AD.

**FIGURE 3:**
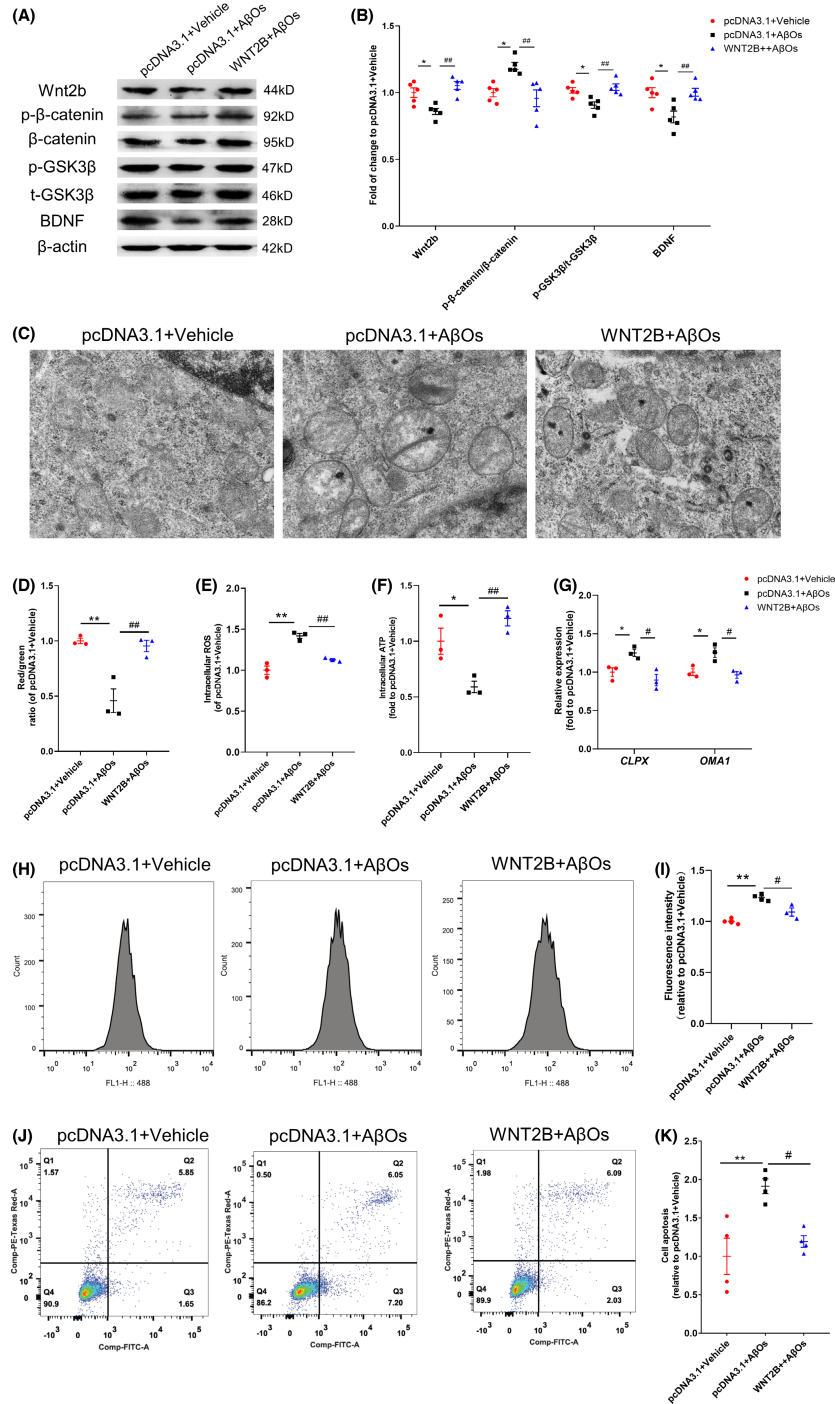
Overexpression of *WNT2B* could prevent mitochondrial dysfunction and neuronal damage induced by Aβ oligomers (AβOs). (A and B) Representative western blots and quantification of fold changes in Wnt2b, p‐β‐catenin, β‐catenin, p‐GSK3β, total p‐GSK3β, BDNF, β‐Actin expression in HT22 cells relative to pcDNA3.1 + vehicle group after AβOs treatment. (C and G) Overexpression of *WNT2B* ameliorated AβOs‐induced disruption of (C) mitochondrial structure and (D) mitochondrial membrane potential, (E) increase of intracellular ROS level, (F) decrease of ATP content and (G) increase of mitochondrial stress gene expression *CLPX*, *OMA1* in HT22 cells. (H and I) Representative flow cytometry and quantification of fold changes in intracellular calcium after AβOs treatment in HT22 cells. (J and K) Representative flow cytometry and quantification of fold changes in neuronal apoptosis after AβOs treatment in HT22 cells. Data values are expressed as mean ± SEM. One‐way ANOVA followed by Tukey's post hoc test as appropriate. *p < 0.05 in comparison to pcDNA3.1 + vehicle, **p < 0.01 in comparison to pcDNA3.1 + vehicle, #*p* < 0.05 in comparison to pcDNA3.1 + AβOs, ##*p* < 0.01 in comparison to pcDNA3.1 + AβOs

Therefore, we explored improving Wnt2b decline on mitochondrial dysfunction and downregulation of canonical Wnt signaling in AD. pcDNA3.1 constructs with *WNT2B* overexpression were used to upregulate the expression of Wnt2b in hippocampal cells, and we used the optimal transfection condition (Figure S3). We found that overexpression of *WNT2B* could significantly reverse increase in p‐β‐catenin /β‐catenin and decrease in p‐GSK3β/GSK3β and BDNF level after AβOs exposure for 24 h (Figure 3A,B). Mitochondrial morphologic was analyzed using electron microscope, we found that the number of mitochondria was decreased and mitochondria showed swelling, disordered ridges, and partial vacuolization in the presence of AβOs, while more mitochondria with arranged ridges were seen in the *WNT2B‐*overexpressed group (Figure 3C **)**. Overexpression of *WNT2B* could effectively ameliorate disruption of mitochondrial membrane potential (Figure 3D, Figure S4), increase in intracellular ROS level (Figure 3E, Figure S4) and decrease in intracellular ATP level (Figure 3F **)** induced by AβOs. Overexpression of *WNT2B* also normalized the increased expression of mitochondrial stress genes *CLPX*, and *OMA1* (Figure 3G).

**FIGURE 4:**
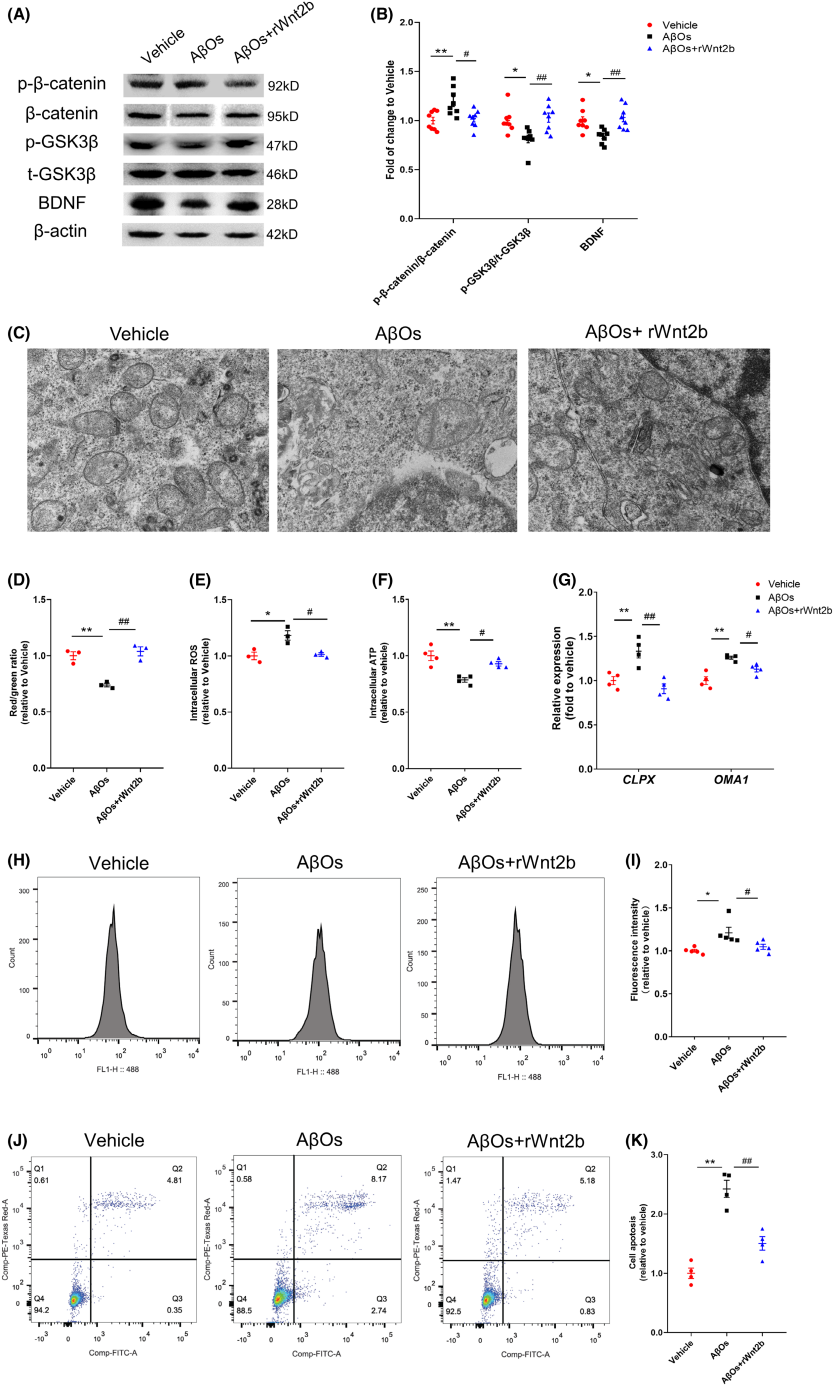
Recombinant Wnt2b (rWnt2b) could prevent mitochondrial dysfunction and neuronal damage induced by Aβ oligomers (AβOs). (A and B) Representative western blots and quantification of fold changes in p‐β‐catenin, β‐catenin, p‐GSK3β, total p‐GSK3β, BDNF, β‐actin expression in HT22 cells relative to vehicle after AβOs or rWnt2b treatment. (C–G) rWnt2b alleviated AβOs‐induced disruption of (C) mitochondrial structure and (D) mitochondrial membrane potential, (E) increase of intracellular ROS level, (F) decrease of ATP content and (G) increase of mitochondrial stress gene expression *CLPX*, *OMA1* in HT22 cells. (H and I) Representative flow cytometry and quantification of fold changes in intracellular Ca^2+^ after AβOs treatment in HT22 cells. (J and K) Representative flow cytometry and quantification of fold changes in neuronal apoptosis after AβOs treatment in HT22 cells. Data values are expressed as mean ± SEM. One‐way ANOVA followed by Tukey's post hoc test as appropriate. *p < 0.05 in comparison to vehicle, **p < 0.01 in comparison to vehicle, #*p* < 0.05 in comparison to AβOs, ##p < 0.01 in comparison to AβOs

Mitochondria play a vital role in calcium homeostasis,^29^ and mitochondrial dysfunction‐induced intracellular calcium imbalance leads to neuronal death and is implicated in AD.^30^ Next, we used flow cytometry to detect the changes in intracellular Ca^2+^ and neuronal apoptosis, and we found that overexpression of *WNT2B* could prevented the increase of intracellular Ca^2+^ (Figure 4H,I) and neuronal apoptosis (Figure 4J,K) in HT22 cells induced by AβOs.

### 3.4 Recombinant Wnt2b rescued mitochondrial dysfunction and neuronal damage in HT22 cells in the presence of AβOs

In addition to endogenously increasing Wnt2b expression in HT22 cells, we tested whether exogenous recombinant Wnt2b (rWnt2b) could ameliorate mitochondrial dysfunction and neuronal damage. Application of rWnt2b (300 ng/mL) significant rescued increase of p‐β‐catenin/β‐catenin, decrease of p‐GSK3β/GSK3βand BDNF level after AβOs exposure 24 hours in HT22 cells (Figure 4A,B). And rWnt2b could alleviate AβOs‐induced mitochondrial swelling, disordered ridge, and partial vacuolization (Figure 4C). rWnt2b could rescue disruption of mitochondrial membrane potential (Figure 4D, Figure S5), increase of intracellular ROS level (Figure 4E, Figure S5), and the decrease of intracellular ATP level (Figure 4E) in HT22 cells. rWnt2b also mitigated the increased expression of mitochondrial stress genes *CLPX* and *OMA1* (Figure 4G). What's more, we found that rWnt2b could prevent the increase of intracellular Ca^2+^ (Figure 4H,I) and neuronal apoptosis (Figure 4J,K) in HT22 cells caused by AβOs.

## 4 DISCUSSION

In the present study, we mainly found plasma Wnt2b levels in AD patients were decreased and positively correlated with cognitive function, and explored the pathological mechanism of Wnt2b in Alzheimer's disease in vitro and in vivo. Interestingly, we found Wnt2b decline was not only involved in downregulation of canonical Wnt signaling but also related to mitochondrial dysfunction and intracellular calcium imbalance in AD. Our findings indicate the potential of Wnt2b in assisting AD diagnosis and provide some reference for AD treatment and interventions.

Previous studies have demonstrated that Wnt2b is required for the development of embryonic forebrain, especially for the lens development.^31^, ^32^, ^33^ Zhang et al. have highlighted the importance of Wnt2b in neurogenesis and found that Wnt2b is related to the particular developing pattern of the fetal hippocampus.^9^ Additionally, *WNT2B* variants contribute to the susceptibility and occurrence of ischemic stroke,^34^ and *WNT2B* is associated with the clinical treatment response and clinical remission in depressed patients, indicating the potential of Wnt2b in neurological disorders.^35^ And our study found Wnt2b decline in AD was associated with cognitive impairment and underlying mechanism for the first time.

In this study, we found Wnt2b decline in the plasma of AD subjects, several studies have suggested that changes in blood Wnt protein can indeed reflect the occurrence and progression of some diseases.^36^ Suzuki et al. have found that some serum peptides, including fragment of Wnt2b, were involved in the pathology of dementia with Lewy bodies (DLB) or Parkinson's disease.^37^ Here, we found plasma Wnt2b levels were lower in AD patients than in age‐matched cognitively normal subjects and Wnt2b is positively correlated with cognitive function, which is consistent with the roles of Wnt signaling on cognition.^38^ Our results provide some reference for Wnt2b in diagnosing neurological diseases including AD. We tried to detect in cerebrospinal fluid, which is more diagnostic than plasma, but cannot be effectively and accurately detected due to insufficient technology and lack of appropriate detection kits. Considering that blood samples are clinically easy to obtain and detect, our study will also provide some help in the diagnosis of AD. We found Wnt2b was reduced in AD animal model and in vitro model. Especially, Wnt2b was found reduced in AD animal model without cognitive impairment at 6‐month‐old, suggesting Wnt2b might play an important role in the early pathogenesis and progression of AD. Studies have reported that there are alterations in biological processes before the onset of AD‐related cognitive impairment.^39^ As to why WNT2B protein and mRNA decreased, we speculated that some non‐coding RNA may affect the decrease of mRNA. Many non‐coding RNAs are altered during the pathogenesis of AD,^40^, ^41^ thus causing changes in some important proteins and genes. Several literatures have reported that miR‐370‐3p, miRNA‐324‐3p, miR‐378a‐3p^42^, ^43^, ^44^ can target, and regulate WNT2B, thereby possibly affecting WNT2B protein and mRNA levels. Besides, epigenetic modifications may also affect WNT2B.^45^ However, we did not investigate the mechanism of  WNT2B protein and mRNA decrease in AD in this study, and we will continue to  explore it. The beneficial effects of *WNT2B* overexpression and recombinant Wnt2b verified the importance of Wnt2b in canonical Wnt signaling and mitochondrial dysfunction in AD. Several lines of evidences hint Wnt2b may play a role in mitochondrial function.^11^, ^12^ We found that Wnt2b was also expressed in mitochondria (Appendix S1 and S2), and aberrant mitochondrial structure and function in AD cell models were improved when *WNT2B* overexpressed or rWnt2b applied, thereby intracellular calcium imbalance and neuronal apoptosis induced by AβOs was prevented. As described in other studies, Wnt signaling is associated with mitochondrial function, particularly in some age‐related disease.^15^, ^46^ Accumulation of damaged and dysfunctional mitochondria has been reported as an early important pathological feature of AD, and further contributes to disease progression.^47^, ^48^ Targeting mitochondria is a promising therapeutic strategy to improve neuronal damage and cognitive impairment in AD.^49^ In the presence of Aβ, abnormal mitochondrial structure and function causes bioenergetic deficiency, intracellular calcium imbalance, and oxidative stress, thereby aggravating the effect of Aβ and tau pathologies, leading to synaptic dysfunction, cognitive impairment, and memory loss.^50^ And disruption of mitochondrial structure and function and mitochondrial stress gene expression was prevented by *WNT2B* overexpression and rWnt2b. Additionally, intracellular calcium accumulation in AD model was ameliorated *WNT2B* overexpression and Wnt2b. Mitochondria is crucial for the maintenance of calcium homeostasis, and dysregulation of mitochondrial calcium homeostasis is related to neuronal cell damage in AD.^51^, ^52^ Knockdown of Wnt expression might decrease biogenesis and induce dysfunction of mitochondria, and trigger cellular senescence.^46^ Wnt signaling is also associated with the regulation of the mitochondrial fission‐fusion process in hippocampal neurons.^53^ Modulating some Wnt protein ligand could prevent the permeabilization of mitochondrial membranes through inhibiting the mitochondrial permeability transition pore or mitochondria from fission‐fusion alterations induced by Aβ.^54^

Wnt2b is an important component of canonical Wnt signaling. In the present study, we found *WNT2B* overexpression and rWnt2b could rescue downregulation of canonical Wnt signaling. The canonical Wnt signaling is widely recognized for improving neuronal damage and cognitive impairment in AD. We consider that the effects of other components in canonical Wnt signaling in addition to Wnt2b on ameliorating mitochondrial dysfunction in in vitro AD model cannot be ignored. β‐catenin has an important role in the maintenance of mitochondrial homeostasis, regulating ATP production via the tricarboxylic acid cycle, OXPHOS, and fatty acid oxidation, which is important for the maintenance of cell function.^55^ Byun et al. found that GSK3 inactivation is importantly and involved in senescence‐associated mitochondrial ROS generation.^56^ Interestingly, decrease in mitochondrial ATP production could specifically downregulate Wnt signaling, and the recovery of the ATP level could restore Wnt signaling activity.^57^ Chen et al. demonstrated that there is a significant positive correlation between BDNF level and mitochondria.^58^ Therefore, we propose that improvements in canonical Wnt signaling and mitochondrial function might be jointly involved in the neuroprotective effect of *WNT2B* overexpression and rWnt2b.

Here, we mainly reported that Wnt2b decline might contribute to neuronal damage by impacting on canonical Wnt signaling and mitochondrial function in AD, while there are still some limitations in the present study. The beneficial effects of overexpression of *WNT2B* or application of rWnt2b were mainly carried out in in vitro AD models and not validated in primary neuronal culture and animal models, which is a shortcoming of this study. We will continue to study the effects of Wnt2b on mitochondrial and synaptic dysfunction and cognitive impairment in primary neurons and animal models in depth. The study mainly detected the mitochondrial structure and function, and specific biological mechanism by which Wnt2b affects also needs to be further studied. What's more, although we detected differences in plasma Wnt2b level between the AD and cognitively normal groups, we will expand the sample size and enroll mild cognitive impairment subjects to further validate the relationship between plasma Wnt2b and cognitive function in AD. Next, we will explore the changes of Wnt2b in CSF and the correlation with plasma Wnt2b by optimizing the detection technology and appropriate kits. And we consider AD is a very complex neurodegenerative disease, and the cognitive diagnosis and assessment of AD should be comprehensive, rather than Wnt2b. If combined with other AD‐related biomarkers, it may be more effective and accurate for the diagnosis of AD.

## 5 CONCLUSION

In summary, the present study mainly found decrease of plasma Wnt2b levels in AD patients, and canonical Wnt signaling and mitochondrial dysfunction might be potential mechanisms for Wnt2b involved in neuronal damage and cognitive impairment in AD. Our findings may advance the understanding of the importance of Wnt family in AD, and we propose that Wnt2b might be a potential diagnostic indicator and therapeutic target for AD.

## AUTHOR CONTRIBUTIONS

Ling‐Zhi Xu and Bing‐Qiu Li equally contributed to the study, and together conducted the majority of the experiments, analyzed the results, and wrote most of the manuscript. Fang‐Yu Li helped with the statistical analysis of the study. Mei‐Na Quan contributed to the language editing of the manuscript. Wei Qin, Ying Li, and Wen‐Wen Li contributed to the cell culture experiments. Yu Zhao contributed to the flow cytometry experiments. Yi‐Ping Wei contributed to the animal experiments. Jian‐Ping Jia contributed to language editing of the manuscript and the supervision of the study work.

## CONFLICT OF INTEREST STATEMENT

The authors declare that they have no conflicts of interest.

## CONSENT TO PARTICIPATE

All participants signed the ethics approval and consent to participate.

## Supporting information


Appendix S1.

Appendix S2.

## Data Availability

The data that support the ﬁndings of this study are available on request from the corresponding author.
