# Supplementary material for: Upregulation of Wnt2b exerts neuroprotective effect by alleviating mitochondrial dysfunction in Alzheimer's disease
Source: CNS Neurosci Ther. 2023 Feb 27;29(7):1805–16. doi: 10.1111/cns.14139 (PMC10324363; doi:10.1111/cns.14139)

Full unedited gel/blot for Figure 1C

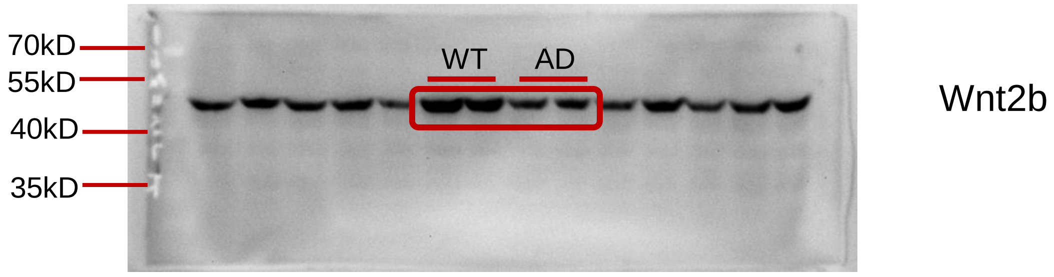

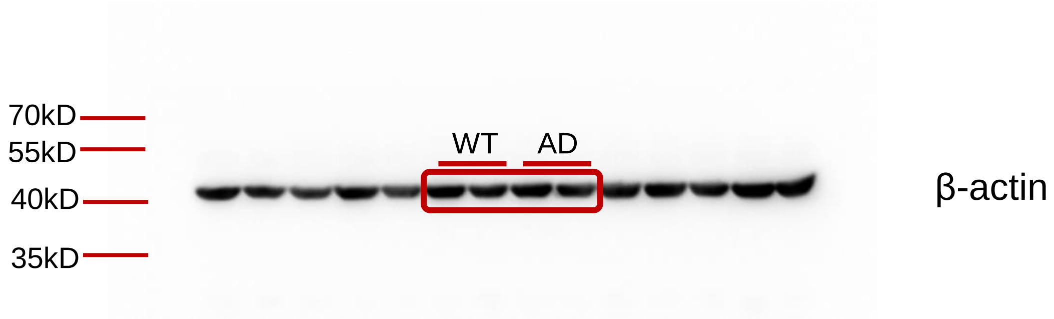

Full unedited gel/blot for Figure 1D

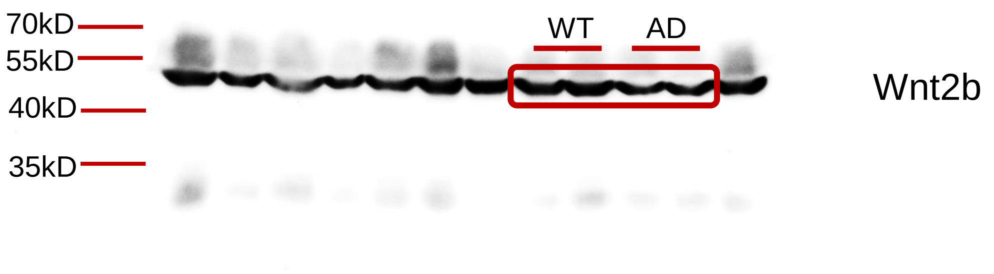

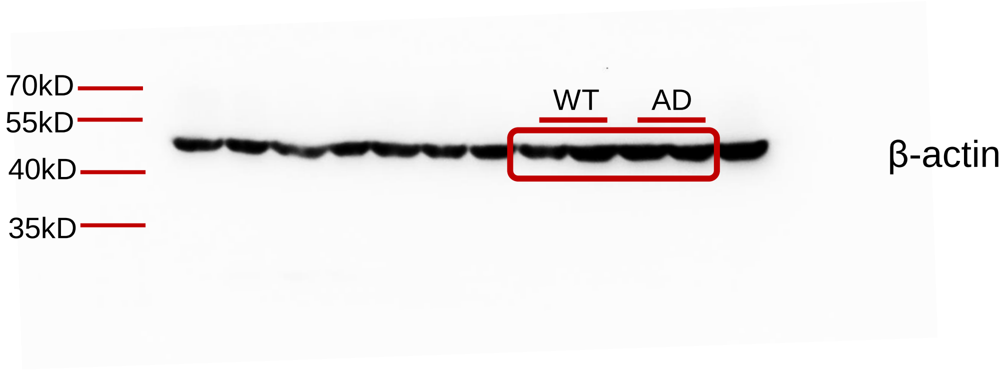

Full unedited gel/blot for Figure 2C

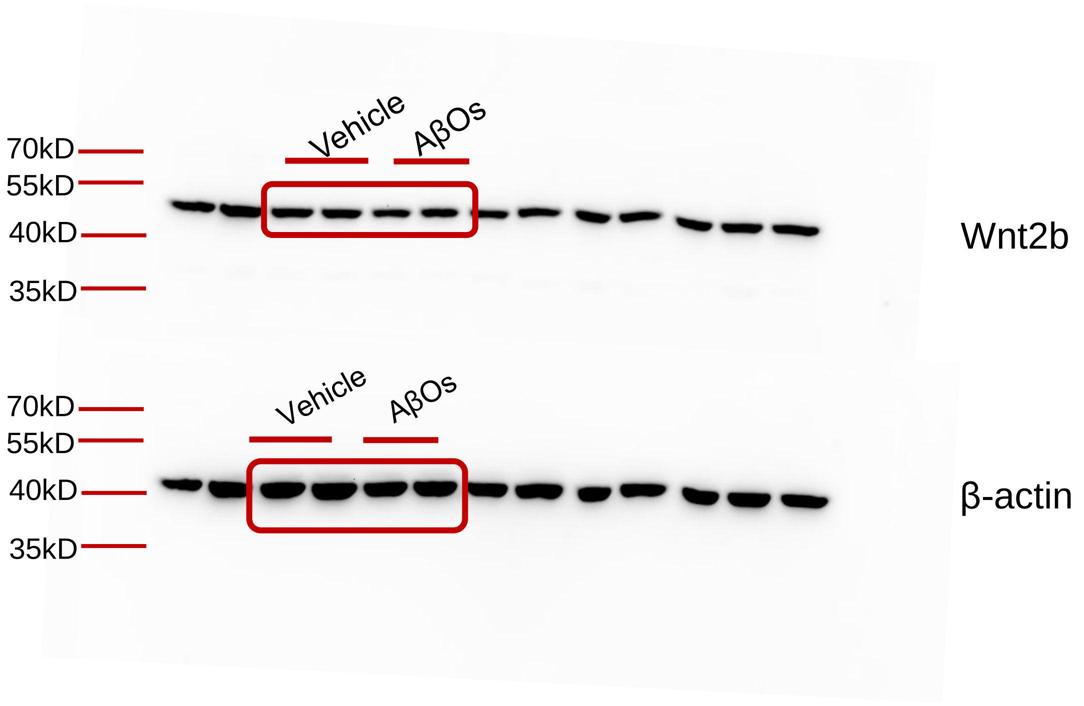

Full unedited gel/blot for Figure 2F

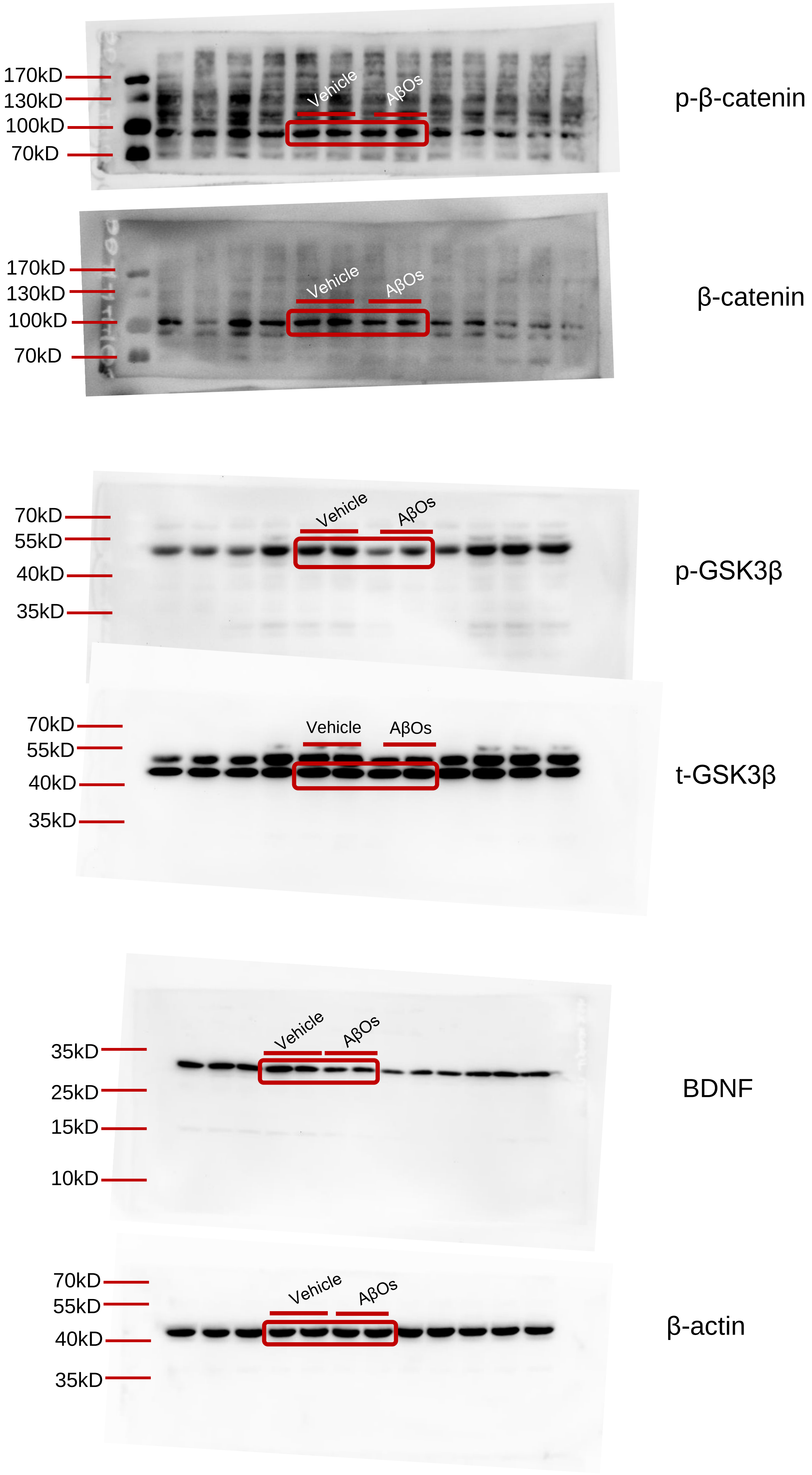

Full unedited gel/blot for Figure 3A

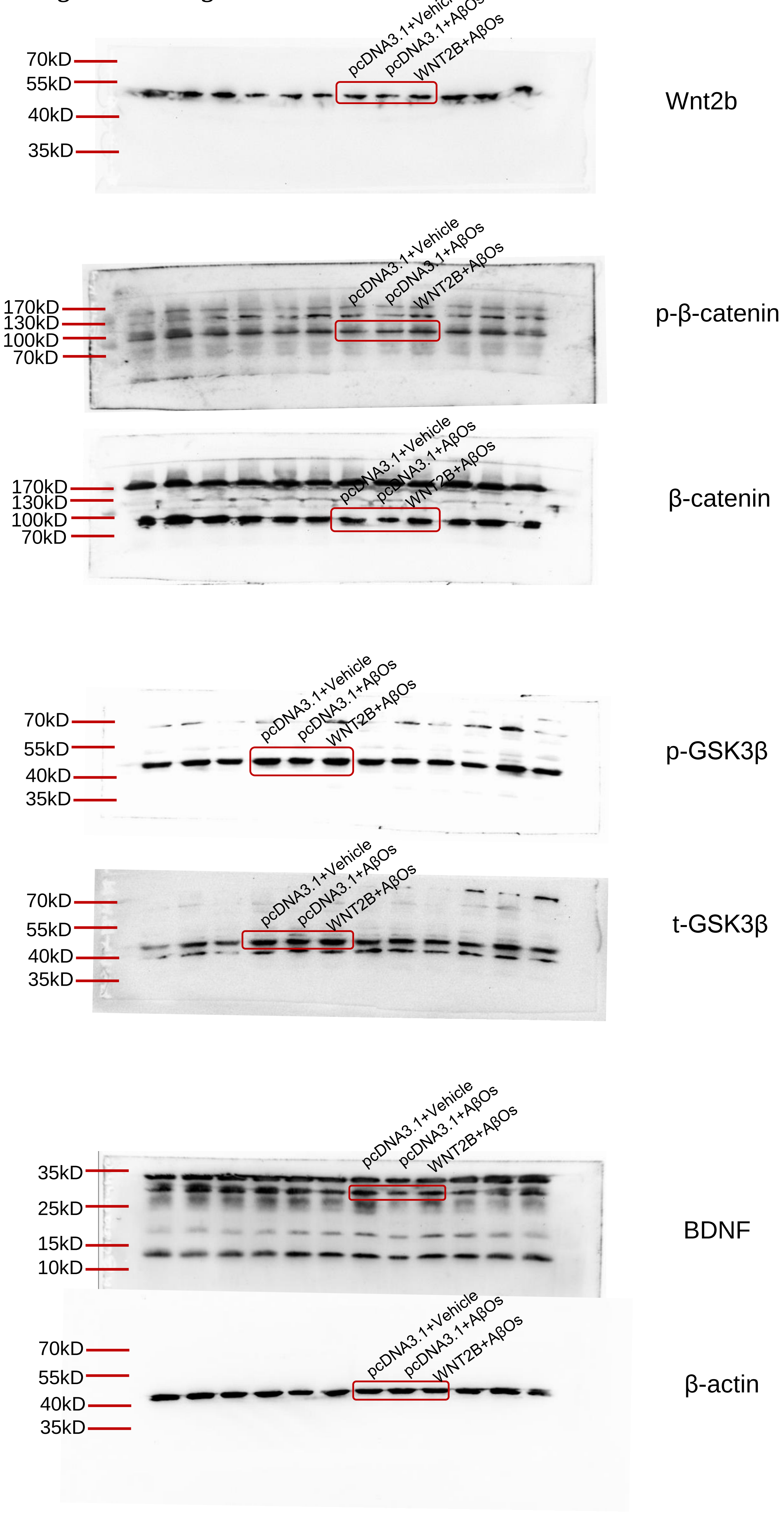

Full unedited gel/blot for Figure 4A

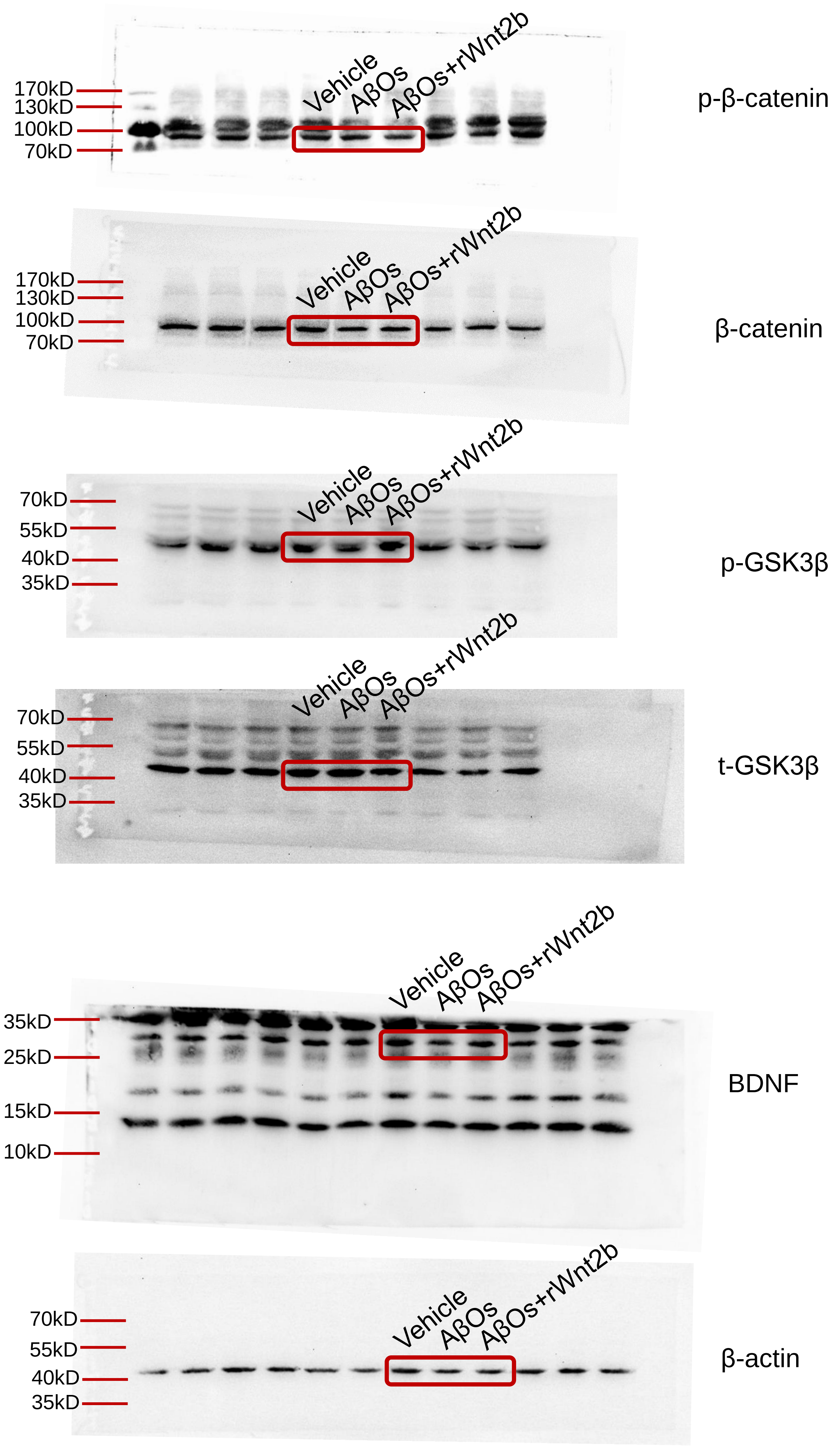

Full unedited gel/blot for Supplementary Figure S2

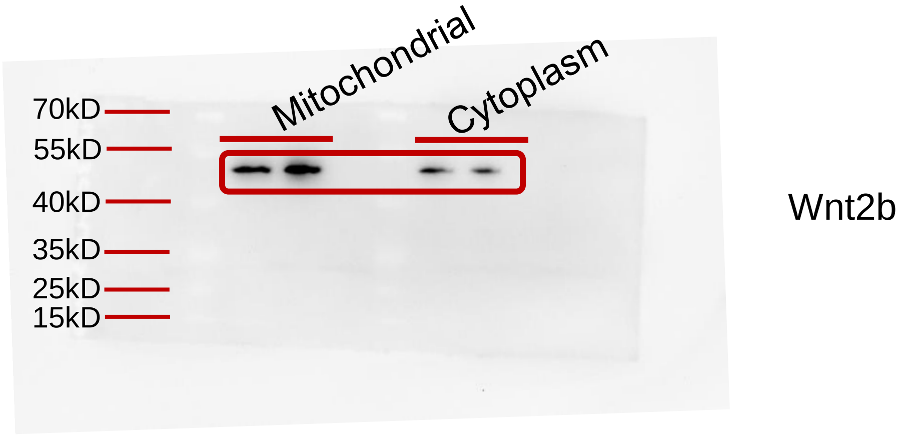

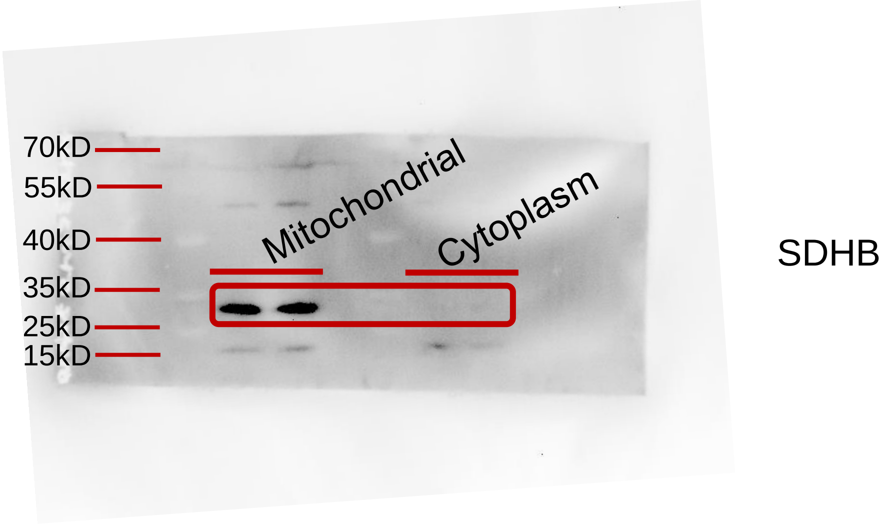

Full unedited gel/blot for Supplementary Figure S3

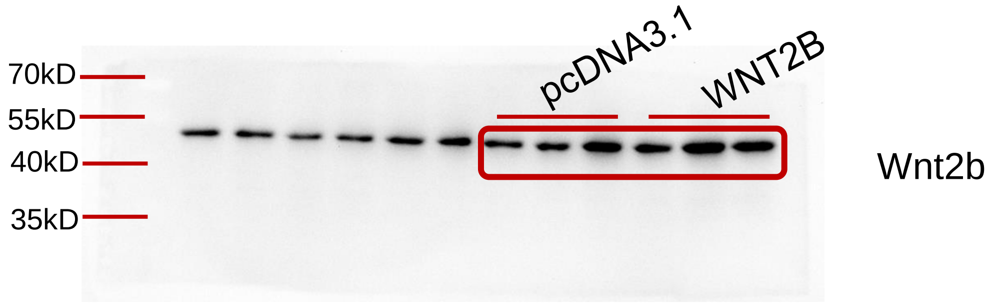

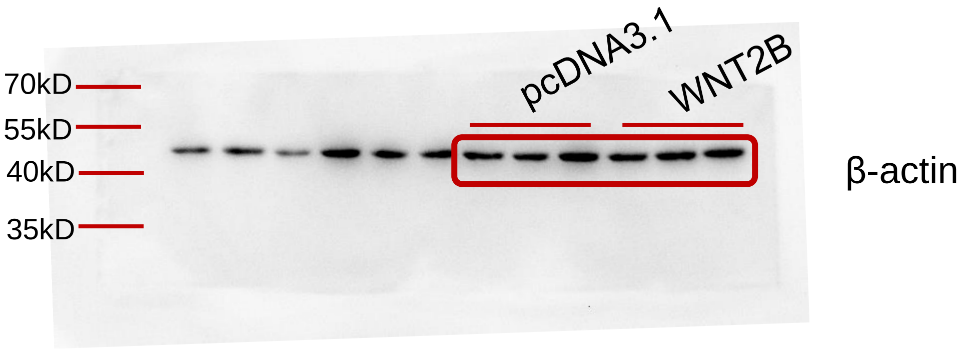

Supplement: Supplementary file 1 — Appendix S1. [file CNS-29-1805-s001.pdf]
