# Supplementary material for: Upregulation of Wnt2b exerts neuroprotective effect by alleviating mitochondrial dysfunction in Alzheimer's disease
Source: CNS Neurosci Ther. 2023 Feb 27;29(7):1805–16. doi: 10.1111/cns.14139 (PMC10324363; doi:10.1111/cns.14139)
Supplement: Supplementary file 2 — Appendix S2. [file CNS-29-1805-s002.docx]

**Supplementary Information**

**Upregulation of Wnt2b exerts neuroprotective effect by alleviating mitochondrial dysfunction in Alzheimer’s disease**

Ling-zhi Xu^1,2,3,4,5†^, Bing-qiu Li^1,2,3,4,5†^, Fang-yu Li^1,2,3,4,5^, Mei-na Quan^1,2,3,4,5^, Wei Qin^1,2,3,4,5^, Ying Li^1,2,3,4,5^, Wen-wen Li^1,2,3,4,5^, Yu Zhao ^5,6^, Yi-ping Wei^1,2,3,4,5^, Jian-ping Jia^1,2,3,4,5*^

1. Innovation Center for Neurological Disorders and Department of Neurology, Xuanwu Hospital, Capital Medical University, National Clinical Research Center for Geriatric Diseases, Beijing P.R. China

2. Beijing Key Laboratory of Geriatric Cognitive Disorders, Beijing P.R. China

3. Clinical Center for Neurodegenerative Disease and Memory Impairment, Capital Medical University, Beijing P.R. China

4. Center of Alzheimer's Disease, Beijing Institute of Brain Disorders, Collaborative Innovation Center for Brain Disorders, Capital Medical University, Beijing P.R. China

5. Key Laboratory of Neurodegenerative Diseases, Ministry of Education, Beijing P.R. China

6. Cell Therapy Center, Beijing Institute of Geriatrics, Xuanwu Hospital Capital Medical University, National Clinical Research Center for Geriatric Diseases

^†^Contributed equally to this work

*Corresponding author: Jianping Jia, MD, PhD, Professor of Neurology, Innovation Center for Neurological Disorders and Department of Neurology, Xuanwu Hospital, Capital Medical University, National Clinical Research Center for Geriatric Diseases, 45 Changchun Street, Xicheng District, Beijing, China, 100053, Tel: +86-10-83199449, E-mail: jpjia@ccmu.edu.cn, [jiajp@vip.126.com](mailto:jiajp@vip.126.com)

**Supplementary Table 1. Results of multiple logistic regression analysis of Wnt2b adjusting for age, gender, *APOE ε4* and education**

|  | B | S.E. | P value | OR (95% CI) |
| --- | --- | --- | --- | --- |
| Constant | 3.041 | 5.45 | 0.577 |  |
| Age | 0.062 | 0.085 | 0.467 | 1.064(0.901-1.256) |
| Gender | -0.084 | 0.659 | 0.899 | 0.92(0.253-3.35) |
| Education | -0.472 | 0.119 | 0 | 0.623(0.494-0.787) |
| *APOE ε4* | 2.669 | 0.858 | 0.002 | 14.426(2.682-77.587) |
| Wnt2b | -0.013 | 0.006 | 0.033 | 0.987(0.976-0.999) |

B: regression coefficient;

S.E.: standard error of regression coefficient;

OR: Odds Ratio


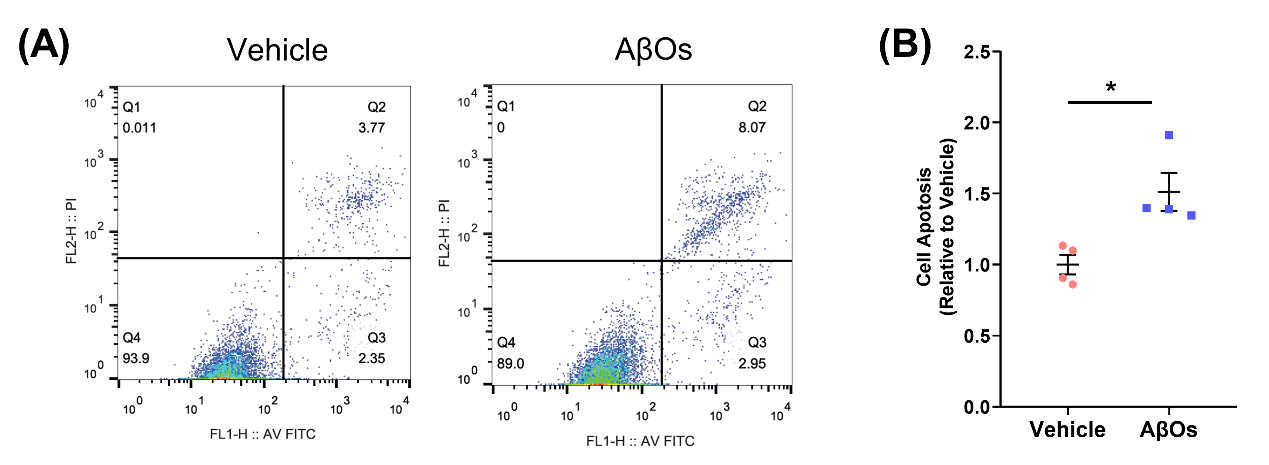


**Supplementary Figure S1** Hippocampal cells showed significant neuronal damage at 24 hours after AβOs exposure. (A) Representative flowcytometry and (B) quantification of fold changes in neuronal apoptosis at 24 hours after AβOs treatment in hippocampal cell. Data values are expressed as mean ± S.E.M. *p < 0.05 in comparison to vehicle.


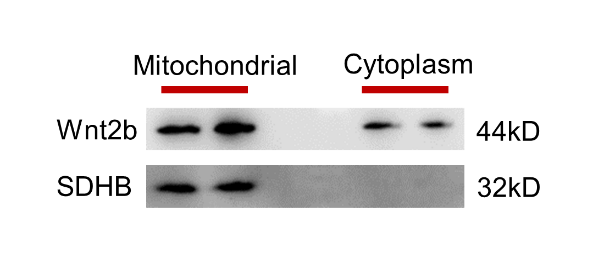


**Supplementary Figure S2** **Representative western blots of Wnt2b and SDHB expression in mitochondria and cytoplasm fraction of hippocampal cell.** SDHB (Succinate Dehydrogenase Complex Iron Sulfur Subunit B), the internal reference of protein from mitochondria.


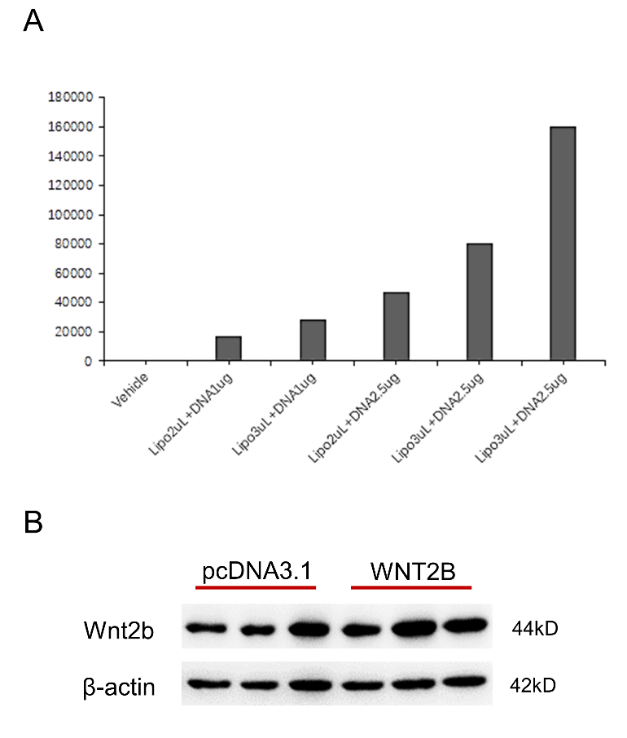


**Supplementary Figure S3** The assessment of optimal transfection condition of WNT2B in hippocampal cell. (A) The transfection efficacy of WNT2B at different concentration of Lipofectamine 3000 and DNA in hippocampal cells was detected by RT-PCR. (B) The transfection efficacy of WNT2B in hippocampal cells was detected by Western blotting.


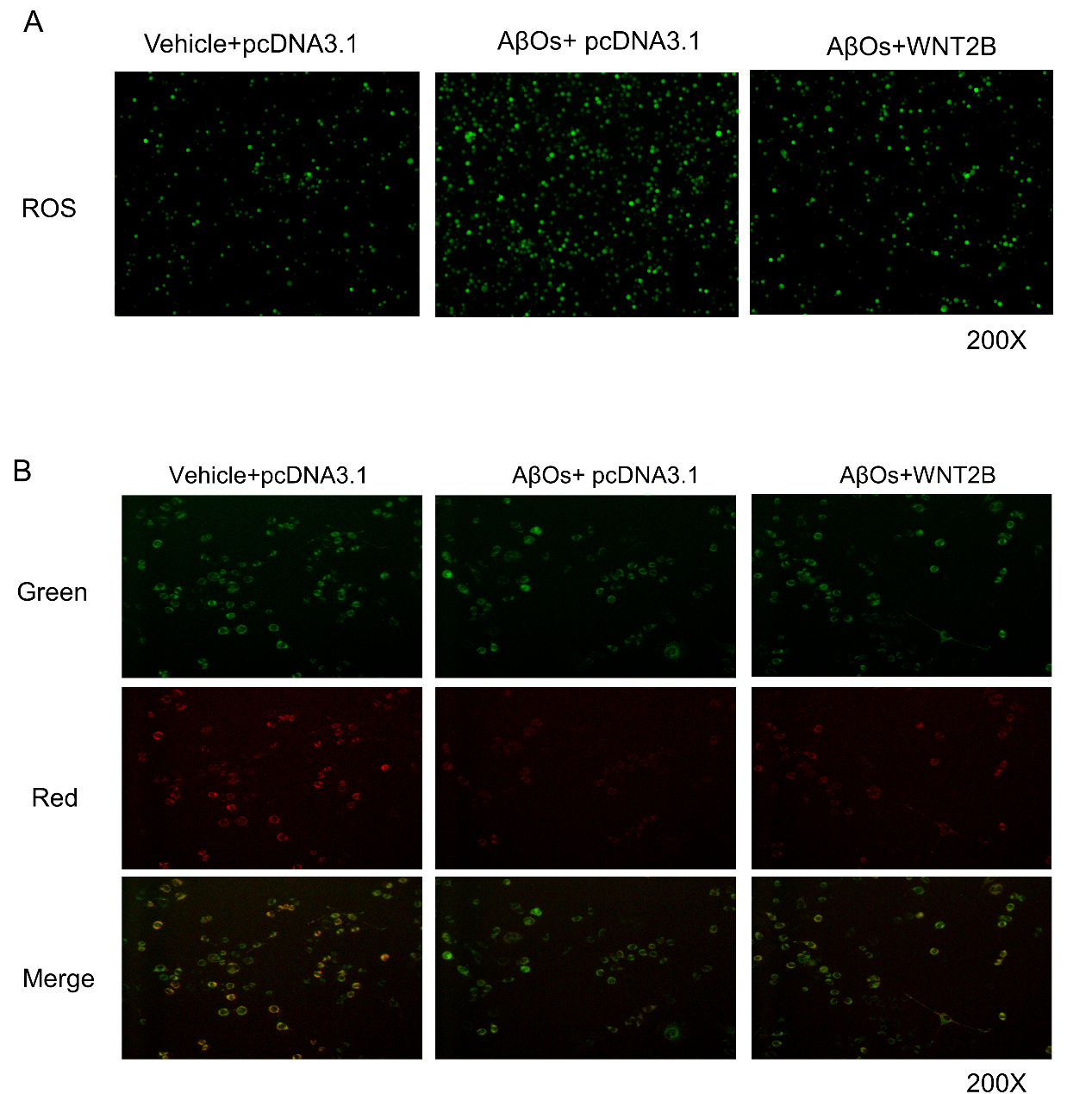


**Supplementary Figure S4** Representative fluorescent image of the effect of overexpression of Wnt2b on intracellular ROS in hippocampal cells. (A) Representative fluorescent image of ROS (green) in hippocampal cells (200X). (B) Representative fluorescent image of JC-1, an indicator of mitochondrial membrane potential in hippocampal cells, green fluorescence represents JC-1 monomer and red fluorescence represents JC-1 aggregate; merged images show combined green and red images (200X).


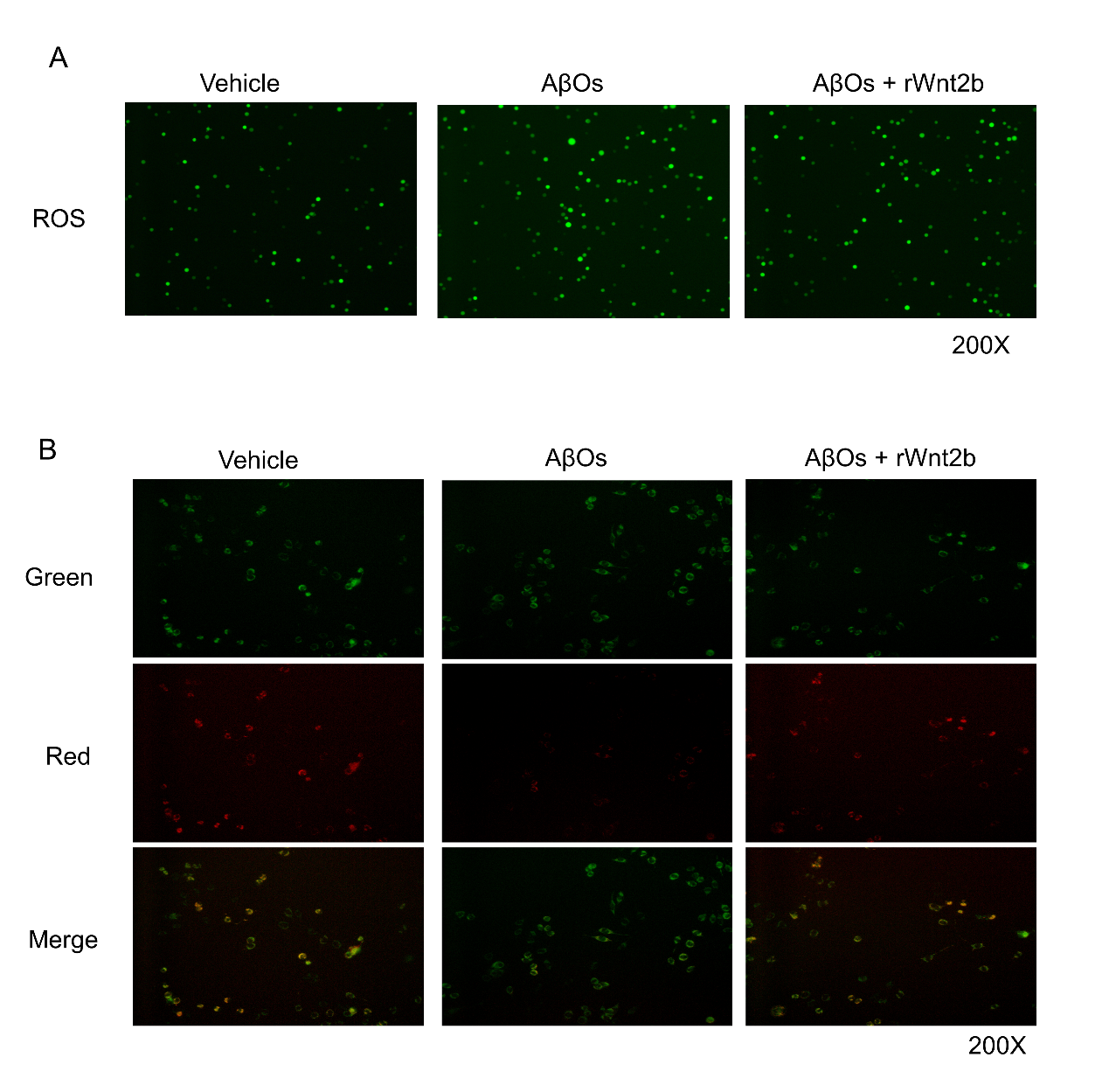


**Supplementary Figure S5** Representative fluorescent image of the effect of recombinant Wnt2b on intracellular ROS and mitochondrial membrane potential in hippocampal cells. (A) Representative fluorescent image of ROS (green) in hippocampal cells (200X). (B) Representative fluorescent image of JC-1, an indicator of mitochondrial membrane potential in hippocampal cells, green fluorescence represents JC-1 monomer and red fluorescence represents JC-1 aggregate; merged images show combined green and red images (200X).
